# Prevalence of Bacterial Pathogens among Symptomatic–SARS-CoV-2 PCR-Negative Patients

**DOI:** 10.3390/microorganisms10101978

**Published:** 2022-10-06

**Authors:** Naveed Ahmed, Saman Habib, Moazza Muzzammil, Ali A. Rabaan, Safaa A. Turkistani, Mohammed Garout, Muhammad A. Halwani, Mohammed Aljeldah, Basim R. Al Shammari, Amal A. Sabour, Maha A. Alshiekheid, Areeg N. K. Abdalla, Jeehan H. Alestad, Saad Alhumaid, Bruno Silvester Lopes, Chan Yean Yean

**Affiliations:** 1 Department of Medical Microbiology and Parasitology, School of Medical Sciences, Universiti Sains Malaysia, Kubang Kerian 16150, Kelantan, Malaysia; 2 Department of Microbiology, Faculty of Science and Technology, University of Central Punjab, Lahore 54000, Punjab, Pakistan; 3 Department of Medical Education, King Edward Medical University, Lahore 54000, Punjab, Pakistan; 4 Molecular Diagnostic Laboratory, Johns Hopkins Aramco Healthcare, Dhahran 31311, Saudi Arabia; 5 College of Medicine, Alfaisal University, Riyadh 11533, Saudi Arabia; 6 Department of Public Health and Nutrition, The University of Haripur, Haripur 22610, Khyber Pakhtunkhwa, Pakistan; 7 Department of Medical Laborator, Fakeeh College for Medical Science, Jeddah 21134, Saudi Arabia; 8 Department of Community Medicine and Health Care for Pilgrims, Faculty of Medicine, Umm Al-Qura University, Makkah 21955, Saudi Arabia; 9 Department of Medical Microbiology, Faculty of Medicine, Al Baha University. Al Baha 4781, Saudi Arabia; 10 Department of Clinical Laboratory Sciences, College of Applied Medical Sciences, University of Hafr Al Batin, Hafr Al Batin 39831, Saudi Arabia; 11 Department of Botany and Microbiology, College of Science, King Saud University, Riyadh 11451, Saudi Arabia; 12 Department of Intensive Care Unit, Saudi German Hospital, Dammam 32313, Saudi Arabia; 13 Immunology and Infectious Microbiology Department, University of Glasgow, Glasgow G1 1XQ, UK; 14 Microbiology Department, College of Medicine, Jabriya 46300, Kuwait; 15 Administration of Pharmaceutical Care, Al-Ahsa Health Cluster, Ministry of Health, Al-Ahsa 31982, Saudi Arabia; 16 School of Health and Life Sciences, Teesside University, Middlesbrough TS1 3BA, UK; 17 National Horizons Centre, Teesside University, Darlington DL1 1HG, UK

**Keywords:** respiratory infections, COVID-19 negative, symptomatic patients

## Abstract

The epidemiological and clinical aspects of coronavirus disease-2019 (COVID-19) have been subjected to several investigations, but little is known about symptomatic patients with negative SARS-CoV-2 PCR results. The current study investigated patients who presented to the hospital with respiratory symptoms (but negative SARS-CoV-2 RT-PCR results) to determine the prevalence of bacterial pathogens among these patients. A total of 1246 different samples were collected and 453 species of bacterial pathogens were identified by culture. Antibiotic susceptibility testing was performed via the Kirby Bauer disc diffusion test. Patients showed symptoms, such as fever (100%), cough (83%), tiredness (77%), loss of taste and smell (23%), rigors (93%), sweating (62%), and nausea (81%), but all tested negative for COVID-19 by PCR tests. Further examinations revealed additional and severe symptoms, such as sore throats (27%), body aches and pain (83%), diarrhea (11%), skin rashes (5%), eye irritation (21%), vomiting (42%), difficulty breathing (32%), and chest pain (67%). The sum of *n* = 1246 included the following: males, 289 were between 5 and 14 years, 183 (15–24 years), 157 (25–34 years), 113 (35–49 years), and 43 were 50+ years. Females: 138 were between 5 and 14 years, 93 (15–24 years), 72 (25–34 years), 89 (35–49 years), and 68 were 50+ years. The Gram-positive organisms isolated were *Staphylococcus aureus* (*n* = 111, 80.43%, MRSA 16.6%), *E. faecalis* (*n* = 20, 14.49%, VRE: 9.4%), and *Streptococcus agalactiae* (*n* = 7, 5.07%), while, Gram-negative organisms, such as *E. coli* (*n* = 135, 42.85%, CRE: 3.49%), *K. pneumoniae* (*n* = 93, 29.52%, CRE: 1.58%), *P. aeruginosa* (*n* = 43, 13.65%), *C. freundii* (*n* = 21, 6.66%), *Serratia* spp. (*n* = 8, 2.53%), and *Proteus* spp. (*n* = 15, 4.76%) were identified.

## 1. Introduction

Respiratory tract infections (RTIs) are infectious diseases that affect the respiratory system [1]. These infections are usually classed as either upper respiratory tract infections (URTIs) or lower respiratory tract infections (LRTIs) [2,3,4]. Infections of the respiratory system include infections of the lungs, pleural cavity, bronchial tubes, trachea, upper respiratory tract, and the nerves and muscles utilized in breathing, either separately or in combination. In various settings, respiratory disorders range from minor to life-threatening [5]. Tonsillitis, pharyngitis, laryngitis, sinusitis, otitis media, certain influenza strains, and the common cold are examples of upper respiratory tract diseases [6,7]. Respiratory infections frequently follow distinct seasonal patterns, with temperate climates being more affected during the winter [8,9]. 

URTIs continue to have critical impacts on society (both economically and socially). The most common bacterial infections are caused by *Streptococcus pyogenes*, *Staphylococcus aureus*, *Acinetobacter baumannii*, *Pseudomonas aeruginosa*, *E. coli*, *Klebsiella pneumoniae*, *Serratia* species, and *Citrobacter* species [10,11,12]. Smokers, children, the elderly, HIV/AIDS-positive individuals, and people with asthma fall into the high-risk group prone to URTIs [13].

In suspected cases of COVID-19, detection of other pathogens is often delayed until the COVID-19 status is confirmed by RT-PCR. Cultures for bacterial pathogens may be delayed; this has further implications as targeted therapy cannot begin. Effective empirical therapy can only be administered if the managing clinicians know the possible respiratory pathogens, which may differ during the outbreak period. This study offers epidemiological insight into circulating pathogens, which not only enable the formulation of more effective empirical therapy but also provide a better understanding of the steps needed in order to improve the patient’s health.

## 2. Materials and Methods

### 2.1. Ethical Consideration 

Ethical approval from the human research ethics committee of the University of Central Punjab, Lahore, Pakistan, was obtained before starting the research. The concerned patient or any individual associated with that patient was required to provide written consent. The pre-determined sheet for data collection also included recommended antibacterial drugs, comorbidities, the total number of provided antibacterial drugs, and demographic data, such as gender and age.

### 2.2. Sample Collection 

The current study was conducted between 3 November 2021 and 14 June 2022 on patients who were COVID-19 PCR-negative but critically ill with pneumoniae-like symptoms. The study was conducted at a tertiary care hospital in Lahore, Pakistan, with a capacity of around 1650 beds. The patients presented to the hospital with COVID-19-like symptoms and were first tested for COVID-19. The patients with COVID-19 PCR-negative results were included in the current study to further investigate the possibility of bacterial infections. To study a more diverse group of patients, and keeping in mind the testing facility, each patient proceeded with one type of sample only. Different types of samples were collected from suspected patients, including blood (*n* = 218) (collected in Thermo Scientific™ Oxoid™ Signal™ Blood Culture System/vials), sputa (*n* = 183), nasal swabs (*n* = 58), throat swabs (*n* = 58), tracheal aspirates (*n* = 253), bronchial alveolar lavages (*n* = 188), pus (*n* = 98), wound swabs (*n* = 106), and pleural fluids (*n* = 84). For the sample collection, strict standard operating procedures (SOPs) were followed and sterile equipment was used. Samples were immediately transferred to the microbiology laboratory for further processing.

### 2.3. Isolation and Identification of Bacterial Isolates

#### 2.3.1. Inoculation of Bacteria

The specimens were cultured on various media, such as chocolate agar, blood agar, and MacConkey agar (Thermo Fisher Scientific, Inc., Waltham, MA, USA). The blood samples were inoculated on blood agar and MacConkey agar while the throat and nasal swabs were inoculated on blood and chocolate agar. The sputum, tracheal aspirate, bronchoalveolar lavage, pus, wound swabs, and pleural fluid samples were inoculated on blood agar, chocolate agar, and MacConkey agar plates. All chocolate agar-inoculated plates were placed in a candle jar (CO_2_ jar).

In the case of blood cultures, the plates were incubated at 37 °C for 18 to 24 h first and checked for bacterial growth. If no bacterial growth was observed on the plates after 24 h, the samples were reinoculated, incubated, and observed for bacterial growth until the 7th day of sample collection. In the cases of other types of samples, the inoculated plates were observed for 48 and 72 h only.

There were possible chances of >1 type of bacterial infection and the appearance of >1 type of bacterial colony on the agar plates. In this case, if there were > 3 types of bacterial colonies observed on the agar plates, they were considered to be possibly contaminated, and the samples were repeated. In the case of <3 types of bacterial colonies, and these proceeded to the final identification based on the morphology of each isolated bacterial colony.

#### 2.3.2. Microscopic Examination

After the inoculation of samples on various media, every sample underwent a smear preparation on a sterile glass slide and Gram staining to identify the microscopic features of the respective bacteria in the clinical specimens.

#### 2.3.3. Biochemical Identification 

The agar plates that confirmed the growth of bacteria were further used for the identification of bacteria and antibiotic susceptibility patterns. The bacterial assessment was completed in correlation with Gram staining, the observation of bacteria colonies on agar plates, and biochemical identification. Gram-positive bacteria were tested for catalase first; if they were catalase-positive, then they were further tested for DNase, optochin disc, and coagulase. The catalase-negative isolates were processed further for streptococcal grouping and bile esculin testing.

Gram-negative bacteria were examined for lactose-fermenters and non-fermenters first and further confirmed by biochemical tests, such as oxidase, citrate, indole assays, and analytical profile indexing-20E (API-20E) biochemical identification kits (BioMérieux, Marcy-l’Etoile, France). The plates that demonstrated no bacterial growth were labeled “No bacterial growth”.

### 2.4. Antibiotic Susceptibility Profile by the Kirby Bauer Disc Diffusion Test

The AST of the bacteria was conducted using the Kirby Bauer disc diffusion test, and minimum inhibitory concentrations (MICs) were carried out wherever recommended by the guidelines for certain antibiotics (for example, vancomycin). To check the susceptibility patterns, the bacterial colonies were diluted and the turbidity was compared to the 0.5 McFarland standard. It was then spread onto a Muller Hinton (MH) agar plate with the use of a sterile cotton swab and appropriate antibiotic discs. The incubation of plates was carried out at 37 °C for 18 to 24 h. After incubation, the zone where inhibition occurred was determined using a labeled measuring scale. The zone of inhibition was calculated for each antibiotic *v*/*s* pathogen, and results were interpreted in accordance with (CLSI) guidelines 2020 [14,15] for each pathogen. The screenings of methicillin-resistant *Staphylococcus aureus* (MRSA) and methicillin-sensitive *Staphylococcus aureus* (MSSA) were done based on the susceptibility pattern of 30 µg of cefoxitin (FOX) disk diffusion test at 33–35 °C for 16–18 h.

The tested antibiotics for Gram-positive isolates were amikacin (30 µg), ampicillin (10 µg), tetracycline (30 µg), ciprofloxacin (5 µg), tobramycin (10 µg), gentamicin (10 µg), teicoplanin (30 µg), chloramphenicol (30 µg), linezolid (30 µg), cefoxitin (30 µg), clindamycin (2 µg), erythromycin (15 µg), fosfomycin (200 µg), levofloxacin (5 µg), and cotrimoxazole–trimethoprim (23.75 µg). The MIC breakpoints for vancomycin were ≤ 2 µg/mL (sensitive), 4–8 µg/mL (intermediate), and ≥ 16 µg/mL (resistant). Antibiotics used for Gram-negative isolates were amikacin (30 µg), tobramycin (10 µg), gentamicin (10 µg), tetracycline (30 µg), ciprofloxacin (5 µg), levofloxacin (5 µg), amoxicillin–clavulanate (20 µg), ceftriaxone (30 µg), ceftazidime (30 µg), cefepime (30 µg), cefixime (30 µg), cefuroxime (30 µg), cotrimoxazole–trimethoprim (23.75 µg), fosfomycin (200 µg), nalidixic acid (30 µg), imipenem (10 µg), meropenem (10 µg), colistin (10 µg), polymyxin b (300 units), and piperacillin–tazobactam (10 µg).

The zones were calculated in standard unit millimeters (mm). Based on CLSI reference ranges, the results are interpreted as resistant, sensitive, and intermediate [15,16]. The patterns of sensitivity for some of the tested drugs were based on MICs. 

### 2.5. Statistical Analysis 

The final data obtained were saved on a Microsoft Excel data sheet (Microsoft Corporation, Washington, U.S.) and were further processed on SPSS version 26.0 (IBM, New York, NY, USA). The recorded data were further analyzed to calculate percentages, frequencies, confidence intervals (Cl), and probability. The Chi-square test was run to compare the clinical presentations of patients with the severity of patient conditions and the prevalence of bacterial infections among SARS-CoV-2 negative (but symptomatic) patients. A *p*-value < 0.05 was considered statistically significant.

## 3. Results

The age distribution between the male and female groups is presented in Table 1.

Different types of specimens were collected from suspected patients, such as whole blood (*n* = 218, 17%), sputum (*n* = 183, 15%), nasal swab (*n* = 58, 5%), throat swab (*n* = 58, 5%), Tracheal aspirate (*n* = 253, 20%), bronchial alveolar lavage (*n* = 188, 15%), pus (*n* = 98, 8%), wound swab (*n* = 106, 8%), and pleural fluid (*n* = 84, 7%). From these 1246 samples, 412 were found positive for bacterial infections; 41 samples were found to have infections of >1 bacterial type. From the positive bacterial culture samples, 12 samples showed bacterial colonies of 3 different types, while 29 samples showed bacterial colonies of 2 different types. The collective M ± SD based on the clinical isolated specimen was M = 138.4, SD was 72.87, and *p*-value was 0.001.

A total of 453 bacterial species were identified and processed for further identification based on morphology, biochemical examination, and stain-specific isolation tests. The final prevalence of each bacterial isolate is shown in Table 2.

The prevalence of MRSA among *S. aureus* isolates in the current study was 16.6%. while no case of vancomycin-resistant *S. aureus* was found. Vancomycin resistance among *E. faecalis* was found in 5% of isolates. Furthermore, antimicrobial susceptibility patterns for the tested Gram-positive bacterial isolates are presented in Table 3. 

The antimicrobial susceptibility patterns for tested Gram-negative bacterial isolates are presented in Table 4.

All of the patients (*n* = 1246) were clinically examined and recommended for microbiological diagnosis by concerned physicians and consultants based on physical conditions and signs and symptoms. The initial symptoms were fever (100%), cough (83%), tiredness (77%), loss of taste and smell (23%), shaking chills (93%), sweating (62%), and nausea (81%), which are similar to COVID-19 symptoms. However, their COVID-19 PCR screenings were negative. They were tested further for microbiological evaluations based on a few additional and severe symptoms, such as sore throats (27%), aches and pains (83%), diarrhea (11%), skin rashes (5%), eye irritation (21%), vomiting (42%), difficulty breathing (32%), chest pain (67%), and shortness of breath (53%). The detailed descriptions of clinical observations are presented in Table 5. A significant correlation was found between the types of clinical symptoms and susceptibility (*p* < 0.05).

## 4. Discussion

Antibiotics are the preferred medication for treating patients with bacterial infections [17]. It is crucial to utilize the right antibiotics in order to improve patient care, minimize costs, and decrease the duration of hospital stay and emotional burden on families, especially in resource-limited settings. Prior to receiving culture reports, which contain precise information regarding the pattern of antibiotic susceptibility of the suspected organism, patients who are critically ill begin treatments based on antibiograms [18]. Since culture reports take almost 48 h, sometimes based on the conditions of patients, the clinicians cannot wait for the reports, which is why they start treatment based on institutional antibiograms [18]. The current study assessed the pattern of antibiotic susceptibility in bacteria isolated from patients with active signs and symptoms of COVID-19, negative PCR tests, and patients who were treated in tertiary care facilities with specific monitoring. 

Results of the current study showed that the most common infections among the tested population were caused by Gram-negative bacterial isolates. The recent studies from Lahore, Pakistan, examined the prevalence of microbial infections and patterns of AMR in COVID-19 patients in tertiary care hospitals, which revealed that *Pseudomonas aeruginosa* and *Klebsiella pneumoniae* were the most commonly associated bacteria isolated from these patients [19,20,21]. A study from Bangladesh showed a high prevalence of antibiotic-resistant Gram-negative bacteria, such as *Acinetobacter* (46%), *P. aeruginosa* (34.2%), *Proteus* (14%), *Klebsiella pneumoniae* (12%), and *E. coli* (7%), which were most frequently identified [22].

Another study examined the bacteriology of patients and AMR analyses in a tertiary care institution in Ahmedabad, India, which showed that *E. coli* [60.89%] was the most frequently isolated bacteria, followed by *Klebsiella* spp. (14.11%) and *P. aeruginosa* (12.68%) [23]. *Pseudomonas* spp. (29.1%) and *Acinetobacter* spp. (29%) were discovered to be the two most common microorganisms in Jordan [24]. In contrast to these investigations, *E. coli* (41%) was the most common bacterium to be isolated, followed by *Klebsiella pneumoniae* (45%), *Klebsiella freundii* (1.3%), *Citrobacter freundii* (2.1%), *Streptococcus agalactiae* (2.1%), *P. aeruginosa* (16%), and *Serratia liquefaciens* (2.1%) [8].

To determine the prevalence of AMR and antibiotic misuse, and to address the problem of Gram-negative bacterial resistance in patients admitted to tertiary hospitals, a study from Jordan [24] showed that the most prevalent and resistant pathogen in patients was *P. aeruginosa*. Nitrofurantoin, meropenem, and imipenem were more effective against infections caused by *E. coli*. In the current study, it was found that *E. coli* had 84% ampicillin resistance and 88% amoxicillin–clavulanic acid resistance. Meropenem and imipenem were the preferred courses of treatment because *E. coli* has high sensitivity (93%) to these antibiotics. Furthermore, *Proteus* spp. showed the highest resistance (89%) against gentamycin and tetracycline (82%). The *Citrobacter* spp. showed the highest resistance (93%) against ceftriaxone, amoxicillin/clavulanic acid (91%), cefuroxime (90%), and ampicillin (81%). None of the bacterial isolates from the current study showed resistance against polymyxin B, colistin, tigecycline, or tobramycin.

The findings of Kreutz et al., (2020) suggested that *Klebsiella pneumoniae* was 100% ampicillin-resistant and 91% AMC–clavulanic acid-resistant [5]. Gentamicin and amikacin were found to be the preferred treatments of choice. According to a study from India, *Klebsiella pneumoniae* was more sensitive to nitrofurantoin and imipenem as compared to other antibiotics [25]. Results of the current study showed that the *Klebsiella* spp. isolates were 94% amoxicillin–clavulanic acid-resistant and 87% ampicillin-resistant. As mentioned before, none of the isolates showed resistance against polymyxin B, colistin, tigecycline, and tobramycin. Future studies involving a larger sample size and covering multiple centers would be beneficial to understand the pattern of AMR, as well as the relationship between SARS-CoV-2, other respiratory pathogens, and the possibilities of other site bacterial infections.

**Study limitations**: Besides the possibility of bacterial and SARS-CoV-2 infections, the possibility of other respiratory viral infections, or infections by atypical bacteria, were not investigated, which might be important to rule out the possibility of other lethal respiratory infections leading to severe complications. Moreover, due to the single study procedure, a relatively small number of patients were enrolled in the current study. Furthermore, because of the ethical consideration issues from the institution, it was not possible to obtain the patients’ histories of using antibiotics (i.e., with their dosages prior to the emergence of SARS-CoV-2 in 2020). Further large-scale and multi-institutional studies are recommended.

## 5. Conclusions

The most common respiratory diseases include pneumonia, asthma, and chronic obstructive pulmonary disease, which account for 20% to 30% of mortality. The misuse of antimicrobial treatments is a major source of antimicrobial resistance that is leading to failed antibiotic therapies. Broad-spectrum antibiotics affect the gut microbiota and thereby affect the body’s system; they play an important role in the increase of AMR.

## Figures and Tables

**Table 1:** Age-scale of the patients with negative COVID-19 PCR tests.

Age (Years)	Gender		%
	Male	Female	
5–14	113	89	16.2
15–24	183	93	22.1
25–34	157	72	18.3
35–49	289	138	38.6
Above 50	43	69	8.9
**Total**	785	461	1246

**Table 2:** Prevalence of bacterial isolates among the tested patients (*n* = 453).

Bacterial Isolates (*n* = 453)			Total Isolates	%	Cl	*p*-Value
**Gram-positive isolates**	*S. aureus*	MSSA	93	67.3	95%	0.02 *
		MRSA	18	13.04		
	*E. faecalis*	VRE	1	0.72		
		Non-VRE	19	13.76		
	*Streptococcus agalactiae*		7	5.07		
	Total (30.4%)		138	100.0		
**Gram-negative isolates**	*E. coli*	(Non-CRE)	124	39.36		0.03 *
		(CRE)	11	3.49		
	*K. pneumoniae*	(Non-CRE)	88	27.93		
		(CRE)	5	1.58		
	*Pseudomonas aeruginosa*		43	13.65		
	*C. freundii*		21	6.66		
	*Serratia* spp.		8	2.53		
	*Proteus* spp.		15	4.76		
	Total (69.5%)		315	100.0		

MRSA: methicillin-resistant *S. aureus*. MSSA: methicillin-sensitive *S. aureus*. VRE: vancomycin-resistant *Enterococcus*. CRE: carbapenem-resistant *Enterobacteriaceae*. *n*: number. %: percentage. CI: confidence interval. * Significant.

**Table 3:** The antibiotic susceptibility patterns of *Strep. agalactiae*, *S. aureus,* and *Enterococcus faecalis* with reference to CLSI guidelines.

CLSI Drug Panel	Resistance (%)			Susceptibility (%)		
	*Streptococcus agalactiae*	*S. aureus*	*Enterococcus* *faecalis*	*Streptococcus agalactiae*	*S. aureus*	*Enterococcus faecalis*
LEV	85.71	NT	NT	14.28	NT	NT
C	85.71	NT	NT	14.28	NT	NT
E	71.42	91.89	70.0	28.57	8.10	30.0
TE	85.71	NT	NT	14.28	NT	NT
CRO	85.71	NT	NT	14.28	NT	NT
VA	14.28	0	5.0	85.71	100.0	95.0
P	100.0	NT	90.0	0	NT	10.0
AK	NT	12.61	NT	NT	87.38	NT
SXT	NT	74.77	NT	NT	25.22	NT
CIP	NT	NT	80.0	NT	NT	20.0
FOX	NT	16.21	NT	NT	83.78	NT
DA	NT	92.79	NT	NT	7.20	NT
FD	NT	NT	NT	NT	10.81	NT
TOB	NT	85.58	5.0	NT	14.41	95.0
TEC	NT	0	5.0	NT	100.0	95.0
LZD	NT	0	0	NT	100.0	100.0
AMP	NT	93.69	NT	NT	6.30	NT
FOS	NT	NT	55.0	NT	NT	45.0
TGC	NT	NT	15.0	NT	NT	85.0

LEV: levofloxacin. C: chloramphenicol. E: erythromycin. TE: tetracycline. CRO: ceftriaxone. VA: vancomycin. P: penicillin. AK: amikacin. SXT: cotrimoxazole. CIP: ciprofloxacin. FOX: cefoxitin. DA: clindamycin. FD: fusidic acid. TOB: tobramycin. TEC: teicoplanin. LZD: linezolid. AMP: ampicillin. FOS: fosfomycin. TGC: tigecycline. NT: not tested.

**Table 4:** The antibiotic susceptibility patterns of *Klebsiella* spp., *E. coli*, *Proteus* spp., and *Citrobacter* spp. with reference to CLSI guidelines.

CLSI Drug Panel	Resistance (%)				Susceptibility (%)			
	*Klebsiella* spp.	*E. coli*	*Proteus* spp.	*Citrobacter* spp.	*Klebsiella* spp.	*E. coli*	*Proteus* spp.	*Citrobacter* spp.
AMP	87.0	84.4	3	81	12.9	15.4	97	10
AMC	94.6	88.1	7	91	5.3	11.8	97	2
CRO	81.7	45.1	0	93	18.2	54.8	100	6
CXM	81.7	52.5	0	71	18.2	47.4	100	11
CFM	82.7	56.2	0	90	17.2	43.7	100	2
IPM	4.3	6.6	4	0	95.6	93.3	95	100
MEM	5.3	6.6	4	0	94.6	93.3	96	100
TZP	8.6	14.0	8	0	91.3	85.9	84	100
TE	74.1	81.4	82	52	25.8	18.5	15	61
CN	24.7	28.8	89	28	75.2	71.1	3	65
NAL	73.1	84.4	0	NT	26.8	15.4	100	NT
FEP	22.5	28.8	1	27	77.4	71.1	98.5	63
CAZ	17.2	19.2	0	0	82.1	80.7	100	100
PB	0	0	0	0	100	100	100	100
CT	0	0	0	0	100	100	100	100
TGC	0	0	0	0	100	100	100	100
TOB	0	0	0	0	100	100	100	100

AMP: ampicillin. AMC: amoxicillin/clavulanic acid. CRO: ceftriaxone. CXM: cefixime. CFM: cefuroxime. IPM: imipenem. MEM: meropenem. TZP: piperacillin–tazobactam. TE: tetracycline. CN: gentamicin. NAL: nalidixic acid. FEP: cefepime. CAZ: ceftazidime. PB: polymyxin B. CT: colistin. TGC: tigecycline. TOB: tobramycin. NT: not tested.

**Table 5:** Clinical observations of patients with negative COVID-19 PCR tests.

Susceptibility	Clinical Symptoms	*n* = 1246 (%)	Cl	*p*-Value
Common	Fever	100	95%	0.002 *
	Cough	83		
	Tiredness	77		
	Loss of taste or smell	23		
	Shaking chills	93		
	Sweating	62		
	Nausea	81		
Moderate	Sore throat	27		0.01 *
	Aches and pains	83		
	Diarrhea	11		
	Rash on skin	5		
	Irritated eyes	21		
	Vomiting	42		
Severe	Difficulty in breathing	32		0.005 *
	Sweating and shaking chills	12		
	Chest pain	67		
	Shortness of breath	53		

* Significant. CI: confidence interval.

## Data Availability

All of the data related to the current study are reported in the manuscript.
